# Co-modulation of T cells and B cells enhances the inhibition of inflammation in experimental hypersensitivity pneumonitis

**DOI:** 10.1186/s12931-022-02200-9

**Published:** 2022-10-08

**Authors:** Olivier Courtemanche, Carole-Ann Huppé, Pascale Blais Lecours, Ophélie Lerdu, Joanny Roy, Jean-François Lauzon-Joset, Marie-Renée Blanchet, Mathieu C. Morissette, David Marsolais

**Affiliations:** 1 Centre de Recherche de l’Institut Universitaire de Cardiologie et de Pneumologie de Québec - Université Laval, 2725 Chemin Sainte-Foy, Quebec City, QC G1V 4G5 Canada; 2 Department of Medicine, Faculty of Medicine, Université Laval, Quebec City, QC Canada

**Keywords:** Hypersensitivity pneumonitis, Extrinsic allergic alveolitis, S1P_1_, CD69, B cells, Rituximab, Biologics, Adoptive lymphocyte transfer, Conditional knockout

## Abstract

**Background:**

Hypersensitivity pneumonitis (HP) is an interstitial lung disease characterized by antigen-triggered neutrophilic exacerbations. Although CD4^+^ T cells are sufficient for HP pathogenesis, this never translated into efficient T cell-specific therapies. Increasing evidence shows that B cells also play decisive roles in HP. Here, we aimed to further define the respective contributions of B and T cells in subacute experimental HP.

**Methods:**

Mice were subjected to a protocol of subacute exposure to the archaeon *Methanosphaera stadmanae* to induce experimental HP. Using models of adoptive transfers of B cells and T cells in Rag1-deficient mice and of B cell-specific S1P_1_ deletion, we assessed the importance of B cells in the development of HP by evaluating inflammation in bronchoalveolar lavage fluid. We also aimed to determine if injected antibodies targeting B and/or T cells could alleviate HP exacerbations using a therapeutic course of intervention.

**Results:**

Even though B cells are not sufficient to induce HP, they strongly potentiate CD4^+^ T cell-induced HP‑associated neutrophilic inflammation in the airways. However, the reduction of 85% of lung B cells in mice with a CD19-driven S1P_1_ deletion does not dampen HP inflammation, suggesting that lung B cells are not necessary in large numbers to sustain local inflammation. Finally, we found that injecting antibodies targeting B cells after experimental HP was induced does not dampen neutrophilic exacerbation. Yet, injection of antibodies directed against B cells and T cells yielded a potent 76% inhibition of neutrophilic accumulation in the lungs. This inhibition occurred despite partial, sometimes mild, depletion of B cells and T cells subsets.

**Conclusions:**

Although B cells are required for maximal inflammation in subacute experimental HP, partial reduction of B cells fails to reduce HP-associated inflammation by itself. However, co-modulation of T cells and B cells yields enhanced inhibition of HP exacerbation caused by an antigenic rechallenge.

**Supplementary Information:**

The online version contains supplementary material available at 10.1186/s12931-022-02200-9.

## Background

Hypersensitivity pneumonitis (HP) is an interstitial lung disease characterized by flares of neutrophilic inflammation and by a substantial infiltration of lymphocytes in the lung. T cells are at the core of HP pathophysiology. Transfer of antigen-experienced CD4^+^ T cells to naïve mice transfers experimental HP [1], indicating that CD4^+^ T cells are sufficient for pathogenesis. However, prophylactic (i.e. before initiating antigenic exposure) antibody-mediated depletion of CD4^+^ T cells in experimental HP causes modifications of leukocyte populations in the airways but fails to inhibit neutrophilic inflammation [2, 3], suggesting that specific targeting of T cells is challenging and likely insufficient for HP therapy. As underscored by others [4], questions that surprisingly remain unanswered are the distinct contribution and therapeutic amenability of B cells in HP. The relative lack of enthusiasm for B cells may have been fueled by seminal findings such as T cell sufficiency to induce an HP-like disease in mice [1, 5–7], even in the absence of B cells [8]; and by deductive reasoning based on the absence of correlations between antigen-specific circulating antibody levels and severity of HP [9, 10].

Without ignoring the critical contribution of helper T cell-associated type IV hypersensitivity mechanisms in HP [11, 12], the involvement of type III hypersensitivity-related mechanisms cannot be ruled out. B cells are indeed a major lymphocyte subset retained in the HP lung [13, 14]. T and B cells are both seen in the lung parenchyma and their organization into B cell-rich tertiary lymphoid tissues (TLT)s is a histological hallmark of HP [14] that often correlates with the magnitude of airway inflammation [15]. Needless to say, the cascade linking immune complex formation to the complement system, macrophage activation, and the ensuing promotion of airway neutrophilic inflammation in HP is common scientific knowledge [11, 14, 16], and detection of circulating agent-specific antibodies remains a critical aspect of diagnosis and management [17].

Recent case studies and reports suggest that the anti-CD20 biological antibody rituximab, which directly targets B cells and possibly influences T cell-related disease mechanisms, may contribute to improving lung function in chronic HP patients [18–20]. We previously showed (Additional file 1 in [21]) that *Oct co-activator from B cells* (OCA-B)-deficient mice, which feature a combination of impaired B cell maturation, T cell activation, and memory response [22, 23], were protected in the *Methanosphaera stadtmanae (*MSS)-induced HP model. Using the same murine HP model, we then showed that sphingosine-1-phosphate receptor 1 (S1P_1_)-targeting drugs strongly inhibited antigen-induced exacerbation and reduced the accumulation of both B cells and T cells in the airways [14]. We later found that S1P_1_ pharmacological ligands impacted B cell functions other than antibody secretion, such as the production of TNF and T cell cooperation ex vivo [24]. In view that interventions impacting both B cells and T cells appear to efficiently alleviate experimental HP, we decided to probe for the respective contributions of B cells and T cells in subacute experimental HP. We also tested the concept that co-modulating these two cell subsets in pre-established experimental HP may provide stronger inhibition of antigenic exacerbations than modulating them individually.

## Materials and methods

### Animals

C57Bl/6 J, Rag1^tm1Mom^, B6.129P2(C)-Cd19_tm1(cre)Cgn_/J and B6.129S6(FVB)-S1pr1_tm2.1Rlp_/J mice (Jackson Laboratory, ME, USA) were bred and maintained in a specific pathogen-free facility at the Institut Universitaire de Cardiologie et de Pneumologie de Québec-Université Laval. CD19^Cre±^ S1P_1_^loxP+/+^ mice feature CD19-driven deletion of S1P_1_ and CD19^Cre±^ S1P_1_^loxP−/−^ were used as the control strain for results shown in Figs. 2, 3, 4. Considering the early stage of this project, we chose to limit the investigation to female mice (7–9 weeks old) since experimental HP is best characterized in females [14, 25, 26]. Housing, handling, and experimental procedures were approved by the Université Laval Animal protection committee (permit# 2018-063) under the authority and guidelines of the Canadian Council on Animal Care.

### MSS-induced experimental HP

Mice were anesthetized with isoflurane and exposed by intranasal instillation to saline or a suspension of *Methanosphaera stadtmanae* (MSS, 100 µg) in saline three times a week for three to four weeks. The proposed dose and schedule of MSS instillation were chosen based on our previous work showing that it replicates key features of HP/experimental HP upon exposure to the archetypal HP-inducing agent *Saccharopolyspora rectivirgula* [27]. After amplification of MSS under strict anaerobic conditions, cultures were washed, frozen, and lyophilized. The antigenic MSS preparation was made by sonicating lyophilized MSS that was priorly resuspended in sterile saline. Details relating to the timing of MSS rechallenge-induced exacerbations and euthanasia (under ketamine-xylazine) are specified in figure legends. In order to assess subacute inflammation resulting from multiple weeks of MSS exposure, mice were euthanized 24 h after the last MSS instillation, which corresponds to the neutrophilic inflammatory phase. When mice with pre-established HP were rechallenged with a single MSS dose, they were euthanized 48 h after the rechallenge, which corresponds to the peak of granulocyte accumulation in the lung for this specific protocol.

### Lymphocytes isolation and transfer to Rag1-deficient mice

Spleens from C57Bl/6 J mice (Jackson Laboratory, ME, USA) were used to isolate B cells and T cells (purity ≥ 95%) using EasySep Mouse B cell isolation kit, and EasySep Mouse T cell isolation kit (STEMCELL Technologies, Vancouver, Canada). Cells were washed and resuspended in saline for intravenous injection of 5 × 10^6^ B cells and/or 2.5 × 10^6^ T cells to Rag1-deficient mice 2 days prior to initiating MSS exposure.

### Lymphocyte-targeting antibodies injection protocols

Mice underwent the MSS-inducing HP protocol. Forty-eight hours after the last MSS exposure, 0.5 mg of anti-CD19 (Clone 1D3; Bio X Cell) and/or anti-CD4 and anti-CD8 (Clones GK1.5 and 53–6.7; Bio X Cell) or their equivalence in control isotype: IgG2a for anti-CD19 and anti-CD8 (clone 2A3; Bio X Cell) and IgG2b for anti-CD4 (clone LFT-2; Bio X Cell), were solubilised in saline and delivered by intraperitoneal injection. Four days after antibodies injections, mice were rechallenged with MSS or saline and euthanized 48 h later.

### Lung histology and bronchoalveolar lavage fluid (BALF) collection

Lung histology for tertiary lymphoid tissue (TLT) area quantification and BALF collection and analyses were done as described [14]. Briefly, 5 μm-thick coronal slices of 10% formalin-fixed paraffin-embedded left lungs were stained with hematoxylin/eosin. After digitalizing histological preparations, total lung area and the area occupied by TLTs, which were used to compute the percentage of lung area occupied by TLTs, were obtained by manually surrounding structures of interest and deriving pixel areas using the Image J software tools (NIH, MD, USA).

For BALF collection, lungs were washed three times with 1 ml of calcium-free magnesium-free phosphate-buffered saline. Total cell number in BALF was obtained using a hemocytometer on crystal violet-stained cell suspensions. Differential counts were performed on cytospun BALF cells stained with the Hema 3 coloration kit (Thermofisher, Waltham, MA, USA). The absolute numbers of macrophages, lymphocytes, neutrophils, and eosinophils were obtained by multiplying their frequencies with total BALF cell numbers.

### ELISA

For albumin detection, dilute BALFs (1/5000) and standards (7.8–500 ng/ml) were incubated for 1 h at room temperature on mouse albumin capture antibody (Bethyl Laboratories, TX, USA) coated plates. Plates where then washed, and incubated with HRP-coupled anti-albumin whole IgG (Bethyl Laboratories, TX, USA). The TMB substrate was used for spectrophotometric detection. ELISAs for MSS-specific antibodies [27] were performed with serial plasma sample dilutions (from 1:100 to 1:312,500), serial cell-free BALF dilutions (1:5–1:3125), and B cell culture supernatants (1:2). For lymph node B cells antibody detection, mediastinal lymph node (mLN) cells containing 5 × 10^5^ to 10^6^ B cells were plated and incubated for 24 h at 37 °C. Supernatants were deemed positive compared to the experimentally-defined limit of detection obtained using blanks and supernatants from MSS-naïve lymph node cell cultures.

### Flow cytometry

Tissues were processed to single cell suspensions as described [14]. Cell surface labeling was performed for 20 min at room temperature using antibodies targeting B220 (clone RA3-6B2, Biolegend), CD90.2 (clone 30-H12, Biolegend), CD19 (clone 6D5, Biolegend), CD69 (Clone H1.2F3, Biolegend), CD4 (Clone RM4-5, Biolegend) and CD8 (Clone 53-6.7, Biolegend). Fluorescence Minus One (FMO) controls were performed on pooled cells from all experimental groups to assist gating. When median fluorescence intensities (MFI)s were computed, baseline fluorescence obtained with FMO controls was subtracted. Data were acquired using a FACS Diva-driven customized LSR Fortessa (BD Biosciences, NJ, USA) and analyzed using the FlowJo software (Tree Star, OR, USA).

## Statistical analyses

Results are presented using the averages ± SEM. Statistical analyses were performed using one-way or two-way ANOVA, when appropriate, with a Tukey’s multiple comparison post hoc test. When possible, log transformation of the data was used to homogenize inter-group variances when statistically different according to the Brown-Forsythe test. Otherwise, statistical analyses for heteroscedastic data were done using Welch ANOVA with a Dunnett’s T3 comparison post hoc test. The normality of data was confirmed using the Shapiro–Wilk test. The significance threshold was set to p < 0.05.

## Results

### B cells and T cells are required for maximal induction of experimental HP

**Fig. 1:**
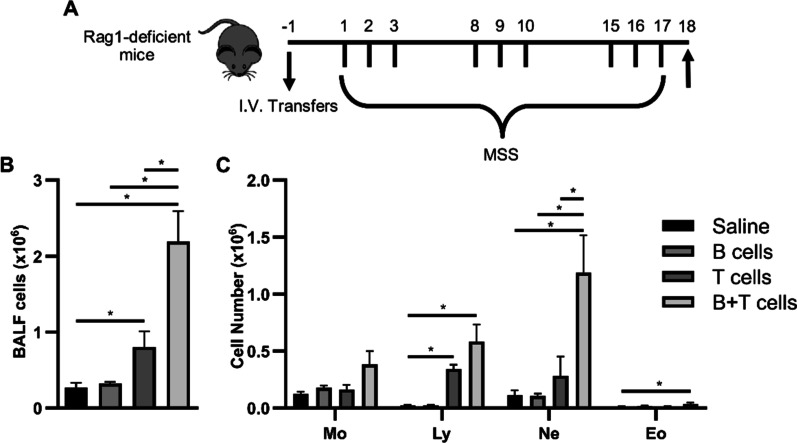
B cells synergize with T cells in the MSS-induced HP model to amplify airway inflammation. **A** Saline, 5 × 10^6^ B cells, 2.5 × 10^6^ T cells, or the combination of 5 × 10^6^ B cells and 2.5 × 10^6^ T cells were injected intravenously to Rag1-deficient mice. Beginning 48 h after the transfer, mice were exposed i.n. three consecutive days a week for three weeks to 100 µg MSS and euthanized 24 h after the last exposure. Upward arrow indicates time of euthanasia. Quantifications of **(B)** total leucocytes and **(C)** differential counts for leucocyte subsets in BALF. I.V.: Intravenous. Mo: Macrophages. Ly: Lymphocytes. Ne: Neutrophils. Eo: Eosinophils. BALF: Bronchoalveolar lavage fluid. MSS: *Methanosphaera stadtmanae*. Averages ± SEM. n = 3–6. *p < 0.05

To directly test the respective contribution of T cells and B cells in the development and intensity of the inflammatory response in HP, Rag1-deficient mice, which feature no mature B cells or T cells, were adoptively transferred with B cells and/or T cells obtained from congenic wild-type mice (Fig. 1A). Rag1-deficient mice that did not receive lymphocytes, or that were priorly injected with B cells featured equally low BALF cell counts (0.27 ± 0.06 × 10^6^ and 0.32 ± 0.02 × 10^6^ respectively) after the HP-inducing protocol (Fig. 1B). Compared to these mice, the HP-inducing protocol caused a twofold increase in average BALF cell numbers when mice were priorly transferred with T cells (0.80 ± 0.21 × 10^6^ cells; Fig. 1B). When mice were transferred concomitantly with B cells and T cells, the HP-inducing protocol caused a strong increase in total BALF cells (2.19 ± 0.39 × 10^6^ cells), which was nearly 3 times higher than in mice transferred with T cells alone (Fig. 1B). Differential BALF cell counts revealed that this enhanced response relied mainly on the increase of neutrophil numbers (Fig. 1C), strengthening the concept that T cells and B cells are required to replicate this central aspect of HP pathogenesis. Noteworthy of mention, transferred T cells and B cells accumulated differentially in the lung vs the spleen at the end of the subacute MSS exposure protocol (Additional file 1: Fig S1). For instance, a robust T cell accumulation was detected in both the lung and the spleen (Additional file 1: Fig S1). In contrast, B cells were nearly undetectable in lung tissue but found in high numbers in the spleen (Additional file 1: Fig S1). Based on these findings, we further addressed whether or not quantitative alteration of B cell accumulation in the lung was sufficient to interfere with HP pathogenesis.

### CD19-driven S1P_1_ deletion inhibits MSS-induced B cell accumulation in the lung

**Fig. 2:**
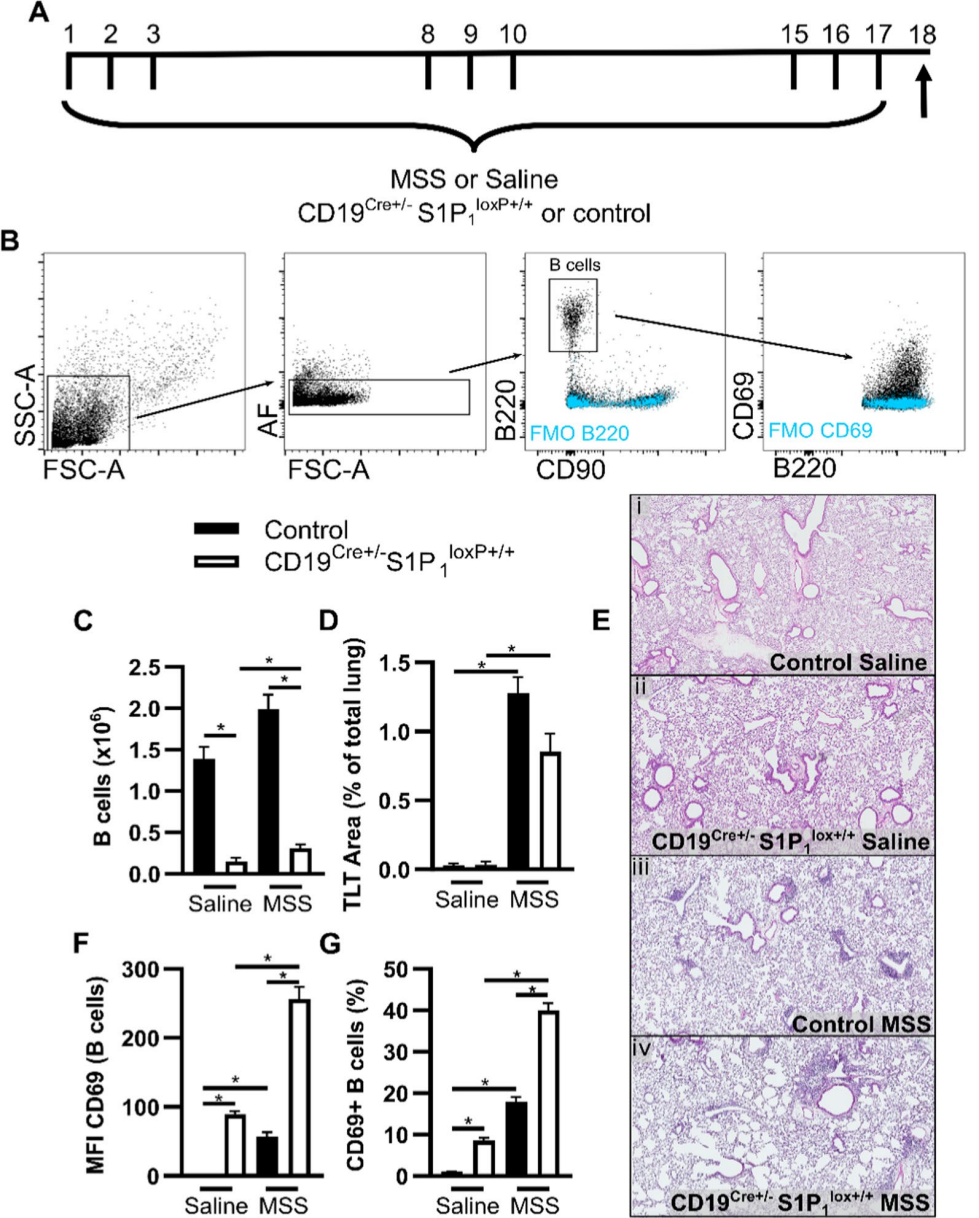
CD19-driven S1P_1_ deficiency inhibits experimental HP-associated B cell accumulation in the lung. **A** Control and CD19^Cre±^ S1P_1_^loxP+/+^mice were exposed three consecutive days a week for three weeks to saline or 100 µg MSS and euthanized 24 h after the last exposure. Upward arrow indicates timing of euthanasia. **B** Gating strategy from single cell suspensions from lungs. FSC-A^low^ SSC-A^low^ cells were selected, and B cells were identified as autofluorescence low (AF^−^) B220^+^CD90^−^ lymphocytes. **C** Frequencies of lymphocyte subsets were multiplied by the total number of lung cells to determine the absolute numbers of B cells. **D** TLT area (percentage of lung area) was quantified as described in the methods after **(E)** hematoxylin/eosin staining of lung coronal slices. **F** CD69 MFI on B220^+^ cells. **G** Frequencies of B220^+^ cells positive for CD69. MFI: median fluorescence intensity. AF: Autofluorescence. TLT: tertiary lymphoid tissue. FMO: Fluorescence Minus One control. MSS: *Methanosphaera stadtmanae*. Averages ± SEM. n = 6–17. *p < 0.05

The sphingosine-1-phosphate receptor S1P_1_ is instrumental for lymphocyte egress from primary and several secondary lymphoid organs [28–30]. Mice lacking S1P_1_ on their B cells (CD19-driven deletion) feature heavily reduced B cell numbers in the blood and peripheral organs [29]. Distinctively from OCA-B-deficient mice, CD19-driven S1P_1_ deletion yields peripheral B cells with an activated phenotype [29]. Mice with CD19-driven S1P_1_ deletion exposed to saline featured low parenchymal lung B cell numbers (0.15 ± 0.04 × 10^6^) compared to the control strain (1.39 ± 0.14 × 10^6^) (Fig. 2A–C). Consistent with previous observations made in wild-type mice [14], the number of B cells was increased by 43% in the lung of mice from the control strain that were exposed to MSS (1.99 ± 0.18 × 10^6^ cells; Fig. 2C). Despite subchronic MSS exposure, total B cell numbers remained very low (0.31 ± 0.04 × 10^6^ cells) in the lung of mice with CD19-driven S1P_1_ deletion (Fig. 2C).

TLTs were nearly absent in the lung of mice exposed to saline (Fig. 2D, E), regardless of the genotype. Exposure to MSS increased the total number of individual TLTs (Fig. 2D, E), which occupied 1.28 ± 0.11% of the total lung area in mice from the control strain. The area of the lung occupied by TLTs was decreased to 0.85 ± 0.13% in mice with CD19-induced S1P_1_ deletion (Fig. 2D, E). This reduction of TLT area is consistent with the profound decrease in B cell numbers, considering the exponential relationship between the cross-sectional area of sphere-like objects and their respective volume.

S1P_1_ and CD69 are mutual counter-regulators [31, 32]. As expected, in the saline-exposed groups, the MFI of CD69 on B cells was higher in mice with CD19-induced S1P_1_ deletion, compared to mice from the control strain (Fig. 2F). MSS exposure increased the MFI of CD69 on B cells isolated from the lung in both strains of mice. CD69 MFI remained higher on B cells from mice with CD19-induced S1P_1_ deletion (256.3 ± 17.8) compared to controls (57.1 ± 6.3) (Fig. 2F). The frequency of B cells expressing CD69 was also higher in mice with CD19-induced S1P_1_ deletion compared to their control strain and this was magnified in the lung in response to MSS (Fig. 2G). We did not observe crucial differences in DC subsets or T cell accumulation/activation in the lung of mice with CD19-induced S1P_1_ deletion in response to MSS (Additional file 1: Fig S2-S3).

### Impact of CD19-driven S1P_1_ deletion on inflammatory hallmarks of MSS-induced HP

**Fig. 3:**
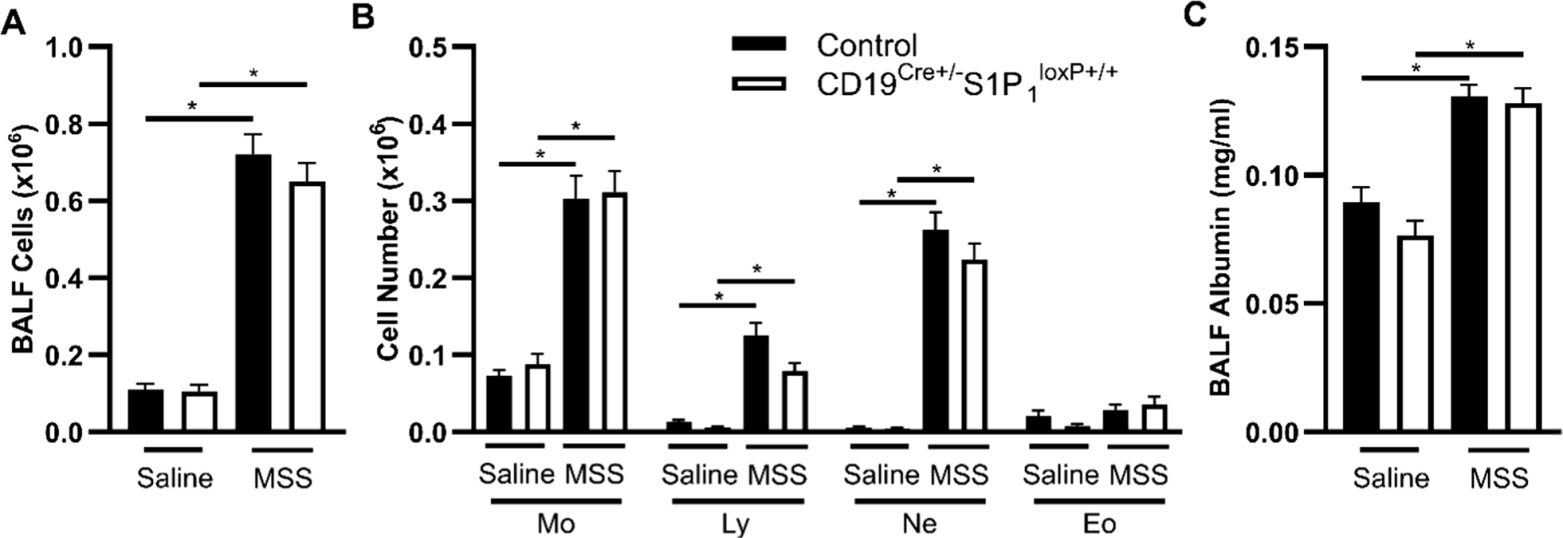
Hallmarks of inflammation in the BALF are unaltered by CD19-driven S1P_1_ deletion. Control and CD19^Cre±^ S1P_1_^loxP+/+^ mice were exposed to either saline or MSS as described in the Methods section and euthanized 24 h after the last MSS instillation. Quantification of **(A)** total leucocytes and **(B)** differential counts for leucocyte subsets in BALF. **C** BALF albumin was quantified by ELISA on BALF supernatant. Mo: Macrophages. Ly: Lymphocytes. Ne: Neutrophils. Eo: Eosinophils. BALF: Bronchoalveolar lavage fluid. MSS: *Methanosphaera stadtmanae*. Averages ± SEM. n = 10–17. *p < 0.05

During the acute phase of experimental HP, B cell numbers and the antigen-specific antibody levels are usually proportional to the extent of neutrophil accumulation and vascular leakage in the airways [33, 34]. In saline-exposed mice, CD19-driven S1P_1_ deletion did not significantly impact BALF cell numbers, nor did it modify the balance between macrophages, lymphocytes, neutrophils, and eosinophils (Fig. 3A, B) when compared to mice from the control strain. In the latter, MSS increased total BALF cells, which was mainly explained by neutrophils and lymphocytes (Fig. 3A, B). Although CD19-driven S1P_1_ deletion trended to decrease BALF lymphocyte counts in response to MSS (0.125 ± 0.016 × 10^6^ for control vs 0.079 ± 0.010 × 10^6^ in mice with CD19-driven S1P_1_ deletion), the accumulation of leukocytes, including neutrophils (Fig. 3A, B), was similar in both strains. The accumulation of albumin in the BALF, a marker of vascular leakage and diffuse alveolar damage [35], also indicated no alleviation of MSS-induced HP exacerbation in mice with CD19-driven S1P_1_ deletion (Fig. 3C). Therefore, the hallmarks of experimental HP, namely neutrophilic inflammation and albumin accumulation in the BALF, were not significantly reduced by CD19-driven S1P_1_ deletion.

### Impact of CD19-driven S1P_1_ deletion on the B cell response to MSS in draining lymph nodes

**Fig. 4:**
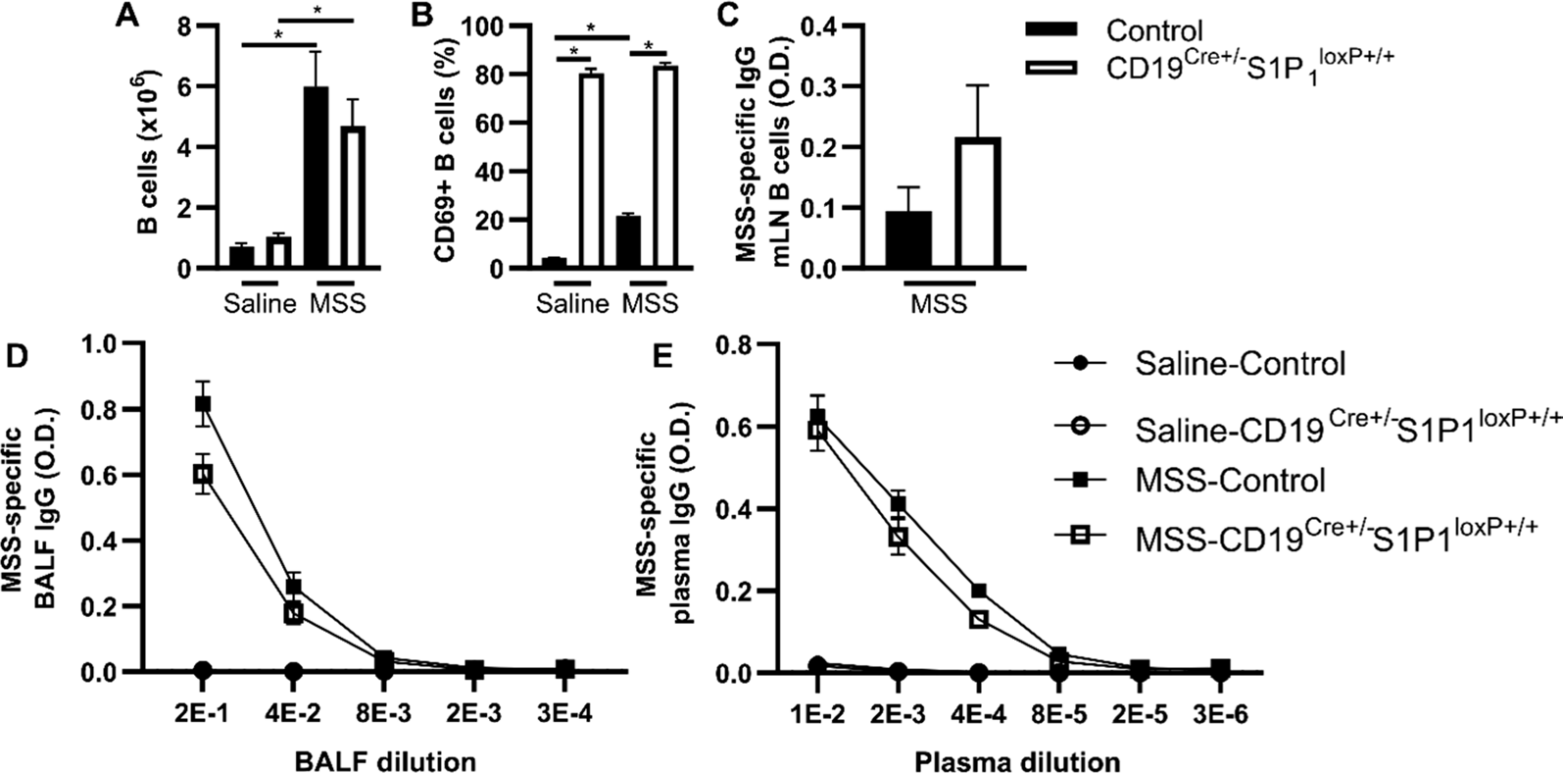
Cells from draining lymph nodes of mice with CD19‑driven S1P_1_ deletion generate MSS-specific IgGs. Mice were exposed to saline or MSS as described in methods and lymph nodes were harvested 24 h after the last instillation. Cytometry was done as described in Fig. 2B to measure **(A)** Numbers of mLN B cells and **(B)** the frequency of CD69^+^ B cells. **C)** MSS-specific IgGs were assessed by indirect ELISA on mLN B cells supernatant diluted 1:2 and on different **(D)** BALF and **(E)** plasma dilutions. mLN: mediastinal lymph node. O.D.: optical density. MSS: *Methanosphaera stadtmanae*. Averages ± SEM. n = 3–6. *p < 0.05

The number of B cells was similar in mLNs of both mouse strains exposed to saline; and subchronic MSS exposure induced a significant B cell accumulation in the mLNs, which again did not differ between the two strains (Fig. 4A). In mice from the control strain exposed to saline, the frequency of CD69^+^ B cells was low (approx. 4%) and increased slightly above 20% in response to MSS (Fig. 4B). On the other hand, the frequency of CD69^+^ B cells was very high (nearly 80%) in mice with CD19-driven S1P_1_ deletion exposed to either saline or MSS (Fig. 4B).

Supernatants of cultured mLN cells retrieved from mice exposed to saline did not contain detectable levels of MSS-specific IgGs when compared to O.D. values from experimental blank samples (i.e. limit of detection). On the other hand, mLN B cell cultures from MSS-exposed mice released detectable amounts of MSS-specific IgGs (Fig. 4C), which occurred regardless of the genotype. We also validated that the critical aspects of the CD4^+^ T cell response in the mLNs (DC and T cell accumulation, activation markers, and polarity) were not significantly altered in mice with CD19-driven S1P_1_ deletion, compared to mice from the control strain (Additional file 1: Fig S4). In line with these findings, plasma and BALF levels of MSS-specific IgGs were similar between control mice and mice with CD19-driven S1P_1_ deletion 24 h after the last exposure to MSS (Fig. 4D, E).

### Combining B cell and T cell-targeting antibodies in pre-established HP inhibits inflammation caused by an antigenic rechallenge

**Fig. 5:**
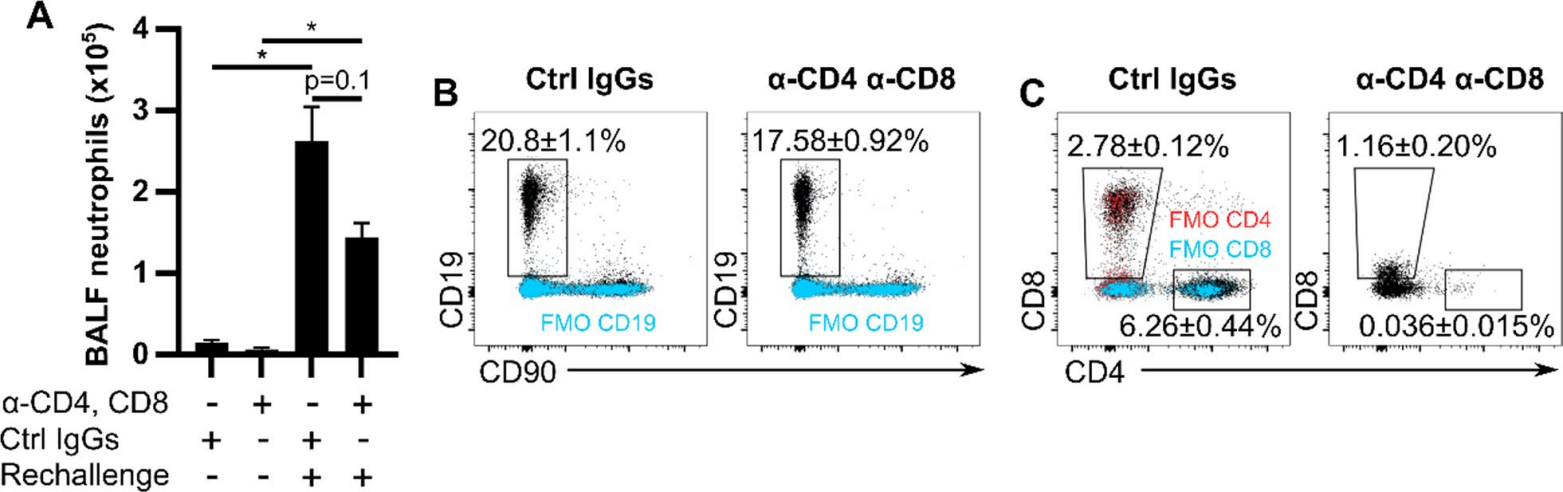
Anti-CD4 and anti-CD8 administration reduces experimental HP inflammation caused by an antigenic rechallenge. C57Bl/6 J mice were administered MSS three times a week for three weeks. Anti-CD4 and anti-CD8 or their respective control IgGs were given intraperitoneally 2 days after the MSS exposure. MSS rechallenge was performed 4 days after antibodies injections and mice were euthanized 48 h later. **A** Quantification of neutrophils/ml of BALF. Single cell suspension from lungs after the MSS rechallenge were analyzed using flow cytometry to determine the frequencies of **(B)** CD19^+^ and **(C)** CD90^+^CD8^+^ and CD90^+^CD4^+^ cells. Fully-labeled specimens are presented in black and FMOs (from pooled cells from all experimental groups) are in blue or red. The percentages shown in **B** and **C** are from fully-labeled specimens. α: anti, MSS: *Methanosphaera stadtmanae*. BALF: Bronchoalveolar lavage fluid, FMO: Fluorescence Minus One control. Averages ± SEM. n = 4–5 *p < 0.05

**Fig. 6:**
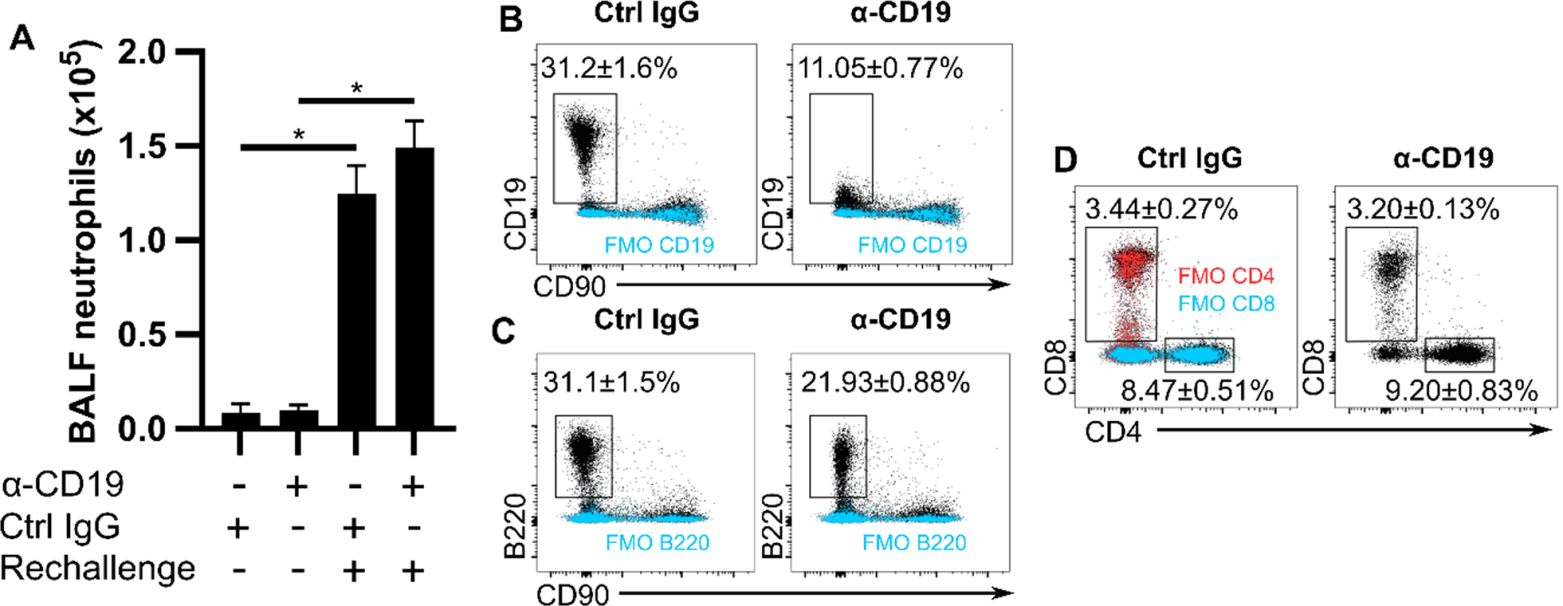
The anti-CD19 does not dampen neutrophilic inflammation caused by an MSS rechallenge in subacute HP. C57Bl/6 J mice were administered MSS three times a week for four weeks. Anti-CD19 or its respective control IgG were given intraperitoneally 2 days after the last MSS exposure. MSS rechallenge was performed 4 days after antibody injection and mice were euthanized 48 h later. **A** Quantification of neutrophils/ml of BALF. Single cell suspensions from MSS-rechallenged lungs were analyzed using flow cytometry to determine the frequencies of **(B)** CD19^+^, **(C)** B220^+^ and **(D)** CD90^+^CD8^+^ and CD90^+^CD4^+^ lung cells. Fully-labeled specimens are presented in black and FMOs (from pooled cells from all experimental groups) are in blue or red. The percentages shown in **B**–**D** are from fully-labeled specimens. α: anti, MSS: *Methanosphaera stadtmanae*. BALF: Bronchoalveolar lavage fluid, FMO: Fluorescence Minus One control. Averages ± SEM. n = 4–6 *p < 0.05

**Fig. 7:**
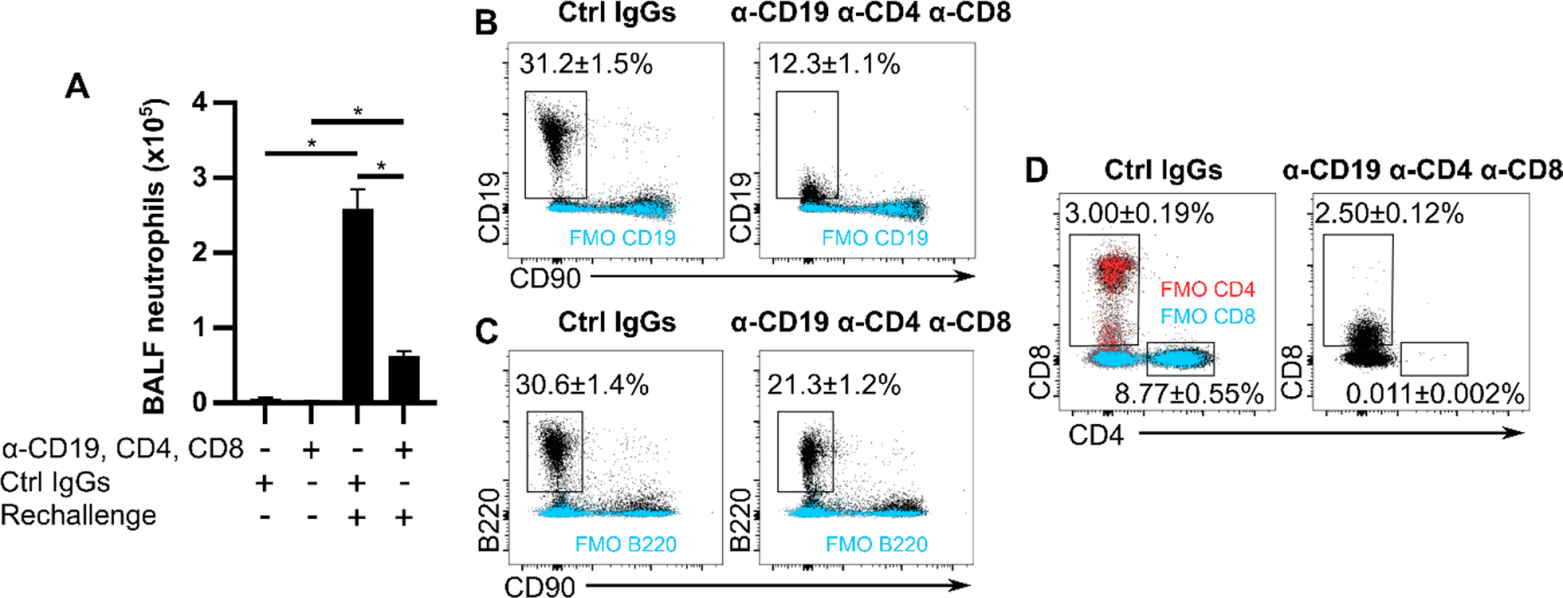
Co-administration of anti-CD19, anti-CD4 and anti-CD8 yields strong inhibition of inflammation caused by an antigenic rechallenge. C57Bl/6 J mice were administered MSS three times a week for four weeks. Anti-CD19, anti-CD4 and anti-CD8 or their respective control IgGs were given intraperitoneally 2 days after the last MSS exposure. MSS rechallenge was performed 4 days after antibody injection and mice were euthanized 48 h later. **A** Quantification of neutrophils/ml of BALF. Single cell suspensions from MSS-rechallenged lungs were analyzed using flow cytometry to determine the frequencies of **(B)** CD19^+^, **(C)** B220^+^ and **(D)** CD90^+^CD8^+^ and CD90^+^CD4^+^ cells. Fully-labeled specimens are presented in black and FMOs (from pooled cells from all experimental groups) are in blue or red. The percentages shown in **B**–**D** are from fully-labeled specimens. α: anti, MSS: *Methanosphaera stadtmanae*, BALF: Bronchoalveolar lavage fluid. FMO: Fluorescence Minus One control. Averages ± SEM. n = 4–5 *p < 0.05

To test the concept that co-modulation of T cells and B cells once experimental HP is established could alleviate the inflammatory response to an antigenic re-encounter, mice were injected i.p. with antibodies directed against CD8, CD4, and/or CD19 after they were exposed to MSS three times a week for three or four weeks (see methods for clones and details). Four days after injection of antibodies, mice were re-exposed to MSS and euthanized 48 h later to investigate the neutrophilic inflammation. When a combination of anti-CD4 and anti-CD8 was used (Fig. 5), the MSS rechallenge-induced neutrophilic inflammation was cut by 45% compared with mice receiving control immunoglobulins (2.62 ± 0.42 × 10^5^ for the isotypic control group vs 1.44 ± 0.18 × 10^5^ for the anti-CD4 and anti-CD8 group; Fig. 5A). Contrarily to T cell-targeting antibodies, anti-CD19 injection did not reduce MSS rechallenge-induced neutrophilic inflammation when compared to its matched isotype-controlled group (Fig. 6A). However, the strongest inhibition of neutrophilic inflammation following the rechallenge occurred when anti-CD19, anti-CD4, and anti-CD8 were co-administered. This led to a 76% decrease in neutrophil numbers in the BALF compared with MSS-rechallenged mice that were injected with control IgGs (2.59 ± 0.26 × 10^5^ for the isotypic control group vs 0.62 ± 0.07 × 10^5^ for the anti-CD19, anti-CD4 and anti-CD8 group; Fig. 7A). As evidenced by an alternative pan B cell marker (B220), mice that received anti-CD19 had only partial depletion (approx 30%) of B cells in the lung at the time of euthanasia (Figs. 6C and 7C) even tough anti-CD19 administration considerably reduced detectable CD19 levels (Figs. 6B and 7B). A similar phenomenon was seen for CD8^+^ cells (Figs. 5C and 7D). The anti-CD4 antibody strongly depleted the CD4 T cells (Figs. 5C and 7D), consistently with previous work [36].

## Discussion

Although Schuyler et al., showed that T cells were sufficient to induce HP thirty years ago [1, 2, 5], lymphocyte-targeting therapies only emerged in the late 2010s for this class of disease. HP manifestations and clinical management evolved in the last decades. Upon identification of etiological agents, it became clear that antigen avoidance efficiently resolved acute symptoms and halted HP progression [9]. Nowadays, the proportion of patients with acute forms of the disease receded but patients with more complex forms, often chronic, for which causal antigens frequently are undefined, remain with limited therapeutic options. Classically, corticosteroids were used to manage acute HP exacerbations, but this type of agent appears to have limited impact at ulterior disease stages [37]. More recently, anti-proliferative and lymphocyte-reducing pharmacological agents were tested in HP clinical trials with encouraging, yet limited effects [4, 20], supporting that our understanding of lymphocytes’ involvement in HP pathogenesis and persistence is partial and warrants further investigations.

Contemporary as well as recent findings challenge the idea that the significance of B cells as a therapeutically-amenable target can be deduced from variations of antigen-specific antibody levels. For instance, circulating antibody levels are sustained by numerous mechanisms and, in addition to antibodies’ half-life, may depend on the nature of the causal antigen [38], history of antigenic re-encounter (sometimes unknown), immune status, and medical history [39]. Therefore, the extent of B cells’ contributions to lung inflammation as it is seen in HP needed to be addressed directly, which was accomplished by inducing HP in Rag1-deficient mice adoptively transferred with T cells and/or B cells. Noteworthy of mention, this model replicated previous findings that T cells, but not B cells, are sufficient to induce an HP-like disease [1, 2, 8]; arguing for its biological relevance. Most importantly, it revealed that in our mixed type III-IV hypersensitivity model, the presence of B cells quadrupled the scale of the neutrophilic exacerbation seen with T cells alone, arguing that the pathophysiological relevance of B cell-T cell cooperation greatly exceeds that of T cells by themselves. Our results suggest that co-modulating T cells and B cells may interfere with immune mechanisms involved in the persistence/progression of HP. Nevertheless, our study does not resolve the issue of whether or not this type of co-modulation may slow or reverse HP when it has reached its fibrotic stage.

B cells accumulate in the lung of HP patients and this phenomenon is very well replicated in HP models [14, 34]. Whether or not this phenomenon is required for inducing HP remained unknown. Taking advantage of the CD19-driven S1P_1_ deletion mouse model, we determined that HP could be fully induced in absence of massive B cell accumulation in the lung, arguing against the theory that interventions aimed at reducing B cells locally in the lung may interfere with HP development. Interestingly, B cells in Rag1-deficient mice also did not accumulate prominently in the lung after adoptive transfer, even when administered concomitantly with T cells, strengthening the concept that high levels of lung B cells are not necessary for maximal subacute HP inflammation. It should be noted that mice with a CD19-driven S1P_1_ deletion feature high frequencies of CD69-positive B cells, which notoriously favors retention in lymphoid organs and peripheral tissues. Indeed, CD69 constitutes a key marker of memory B cell homing in the lung [40]. In line with the seminal findings of Allende et al. [29], we found that the frequency of CD69^+^ B cells in the lung was strongly increased upon CD19-driven S1P_1_ deletion. Given the increasing evidence supporting a critical role for CD69 in the retention of antigen-specific B cells and T cells in the airways, and considering that mice with abnormally low B cell numbers that strongly express CD69 appear to develop an HP-like disease in this subacute model, it may be speculated that CD69 could constitute a target of interest in the dysregulated steps leading to chronic HP.

Considering the altered ability of S1P_1_-deficient B cells to recirculate; and since they appear to feature some level of phenotypical modifications [29], we also verified that mice with CD19-driven S1P_1_ deletion were able to mount a specific antibody response to an antigen that was instilled intra-nasally. Although our time-points do not allow for investigating the onset, we found that after weeks of MSS exposure, the levels of circulating MSS-specific IgGs were not reduced in mice with S1P_1_-deficient B cells. Allie et al. [41] determined that memory B cells homing in the parenchyma after airway exposures may never exit the lung, aligning with the classical findings made by Bice and Muggenburg [42] showing that antigen-specific producing B cells may be circumscribed to single lobes. Nevertheless, it was also determined that this anatomically circumscribed antigenic exposure ensued a systemic dissemination of antigen-specific antibodies over time. The proportion of lung vs blood-derived antigen-specific producing B cells was also increased upon multiple airway antigen challenges [43], supporting the possibility that systemic responses may yield quantitatively normal antibody responses in the lung, given enough time. Our finding that mLNs of mice with CD19-driven S1P_1_ deletion feature MSS-specific antibody-producing B cells supports this notion and aligns with the fact that pharmacologically-induced sequestration of lymphocytes in lymphoid organs does not compromise airway pathogen-specific antibody responses [44, 45]. We propose that, in mice with CD19-driven S1P_1_ deletion, circulating MSS-specific antibodies combined with unaltered T cell responses are likely sufficient for HP pathogenesis.

We previously determined that a therapeutic course of treatment with a pharmacological S1P_1_ receptor-ligand reduced lung B cell numbers and experimental HP inflammation after an antigenic rechallenge [14]. We then speculated that reducing B cell numbers in the lung may contribute to alleviating HP inflammation. Nevertheless, our observation that subacute experimental HP occurs in absence of massive B cell accumulation in the airways strongly argues against this theory, which is also refuted by our experiments where anti-CD19 antibodies are injected after experimental HP is already present. CD19 is a crucial co-receptor for B cell receptor (BCR) signaling and is important for primary B cell activation by T cell-dependent antigens as well as memory B cell differentiation [46]. CD19 neutralization likely affects BCR signaling, but blocking CD19 alone in mice with pre-established experimental HP, even though causing a 30% decrease in lung B cell numbers (not shown), did not alleviate inflammation resulting from an antigenic recall. However, co-injection of anti-CD4 and anti-CD8 at the same timepoint diminished by half BALF neutrophil numbers. Although it may be rationalized that this was expected based on T cell’s central role in HP, it should be noted that our findings contrast with those from Denis et al. [3] where injection of anti-Thy1.2, anti-CD8, or anti-CD4 antibodies failed to reduce absolute BALF cell numbers a similar model of HP, the anti-Thy1.2 even causing a surge in neutrophils. However, in the study of Denis et al., antibodies were injected before and during the induction of experimental HP, highlighting that similar T cell-targeting approaches may cause different, even opposite, outcomes at different phases of the disease.

Co-administration of antibodies targeting CD19 and CD4/CD8 produced the greatest decrease in neutrophil recruitment in this series of experiments. These findings suggest that T and B cell cooperation has a preponderant role in HP exacerbations caused by antigen re-encounters. B cell-derived antibodies are thought to be important in the early stages of HP. However, the transfer of antigen-experienced B cells to naïve mice and antigen-specific antibodies containing serum does not transfer HP, contrarily to antigen-experienced T cells [5, 47], supporting the concept that antigen-specific antibodies are not sufficient for HP development. In our therapeutic model using depleting antibodies, B cells were only reduced by 30% and euthanasia was carried out 5 days after depletion. It is likely that MSS-specific antibodies were not potently decreased in mice receiving concomitantly anti-CD19, anti-CD4, and anti-CD8, and thus, not central to the alleviation of inflammation [10].

Other functions of B cells such as antigen presentation to T cells are important in HP development. The removal of splenocytes expressing the MHC class II β Ia^k^ chain prior to adoptive transfer partially protected recipient mice from developing experimental HP [48] and the blockade of CD80/CD86 binding allows to considerably reduce inflammation in response to *Saccharopolyspora rectivirgula* [25]. T cell-B cell cooperation is also known to contribute to cytokine amplification. We showed that pharmacological modulation of S1P_1_ on B cells reduced their ability to induce cytokine production by antigen-specific T cells [24]. A combination of mechanisms thus likely underlies the enhanced inhibition of inflammation when B cells and T cells are concomitantly targeted. Consequently to our experimental design, we cannot however discern respective contribution of the CD4 and CD8 T cell subpopulations to this effect.

Rituximab is sometimes used as a salvage therapy for advanced HP, and it may only benefit subsets of patients [20]. Even though rituximab’s main target is CD20^+^ B cells, it was documented to induce a substantial CD4 T cells depletion in a subset of patients with rheumatoid arthritis, which likely contributed to its clinical efficacy [49]. Our results of enhanced inhibition of antigen-triggered HP exacerbation with dual alteration of T cell and B cell populations argue that a similar phenomenon might apply in the context of HP and might even be at play with emerging HP therapeutics such as mycophenolate mofetil [4], which impacts proliferating cells, preferentially B and T cells.

More than 300 HP causative agents from various sources have been identified, ranging from avian proteins, to bacteria, and fungi among others [50]. Some studies suggest that different HP causative antigens could lead to distinct immune responses [51–53]. However, the main mechanism of action, i.e. type IV hypersensitivity, where CD4^+^ T cells drive an antigen-triggered pro-inflammatory response, seems to be a common feature amongst HP causative agents [50, 53]. Similar to findings made with the commonly employed HP model induced by *Saccaropolyspora rectivirgula*, we determined that the MSS-induced HP model also relies on T cells [1, 8]. In addition, we provide evidence for the notion that B cells are necessary for maximal subacute HP inflammation. The complex interplay between host characteristics, antigen-specific mechanisms, and the different stages of HP is likely to impact key pathognomonic mechanisms, such as T cell polarity, which in turn has defining effects on immunopathological manifestations of the disease [54]. As such, the results of this study might not translate to other classes of antigens or to more chronic HP stages.

Altogether, this study shows that B cells are required for full-scale lung inflammation upon subacute exposure to MSS, but their massive accumulation in the lung is facultative. In the series of experiments where interventions were made after HP was established, co-modulation of B and T cells yielded superior inhibition of inflammation in response to MSS rechallenge compared to modulation of B cells or T cells individually. We speculate that, although T cells are sufficient for inducing HP and B cells are required for maximal inflammation, they may not individually constitute sensible targets for HP therapy.

## Supplementary Information

**Additional file 1.** Additional details relating to leukocyte subset redistribution and activation.** Figure S1**. Adoptively transferred B cells minimally accumulate in Rag1^−/−^ mice lungscontrarily to T cells.** Figure S2**. Unaltered numbers of macrophages and DCs in the lung of mice with CD19-drivenS1P_1_ deletion.** Figure S3**. Effect of CD19-driven S1P_1_ deletion on archetypal HP-associated CD4 T cell responsesin the lung.** Figure S4**. Impact of CD19-driven S1P_1_ deletion on mLN DC and CD4 T cells.

## Data Availability

The datasets used and/or analysed during the current study are available from the corresponding author on reasonable request.

## Abbreviations

BALF: Bronchoalveolar lavage fluid
BCR: B cell receptor
HP: Hypersensitivity pneumonitis
FMO: Fluorescence minus one
MFI: Median fluorescence intensity
mLN: Mediastinal lymph node
MSS: *Methanosphaera stadtmanae*
OCA-B: Oct co-activator from B cells
S1P_1_: Sphingosine-1-phosphate receptor 1
TLT: Tertiary lymphoid tissue

## References

[1] Schuyler M, Gott K, Shopp G, Crooks L (1992). CD3+ AND CD4+ cells adoptively transfer experimental hypersensitivity pneumonitis. Am Rev Respir Dis.

[2] Schuyler M, Gott K, Edwards B, Nikula KJ (1994). Experimental hypersensitivity pneumonitis - effect of CD4 cell depletion. Am J Respir Crit Care Med.

[3] Denis M, Cormier Y, Laviolette M, Ghadirian E (1992). T cells in hypersensitivity pneumonitis: effects of in vivo depletion of T cells in a mouse model. Am J Respir Cell Mol Biol.

[4] Morisset J, Johannson KA, Vittinghoff E, Aravena C, Elicker BM, Jones KD, Fell CD, Manganas H, Dube BP, Wolters PJ (2017). Use of mycophenolate mofetil or azathioprine for the management of chronic hypersensitivity pneumonitis. Chest.

[5] Schuyler M, Gott K, Shopp G, Crooks L (1993). CD3+, CD4+, CD8-, IA- T-cells adoptively transfer murine experimental hypersensitivity pneumonitis. Chest.

[6] Schuyler M, Gott K, Cherne A, Edwards B (1997). Th1 CD4(+) cells adoptively transfer experimental hypersensitivity pneumonitis. Cell Immunol.

[7] Schuyler M, Gott K, Edwards B (1999). Th1 cells that adoptively transfer experimental hypersensitivity pneumonitis are activated memory cells. Lung.

[8] Bernatchez E, Langlois A, Brassard J, Flamand N, Marsolais D, Blanchet MR (2017). Hypersensitivity pneumonitis onset and severity is regulated by CD103 dendritic cell expression. PLoS ONE.

[9] Raghu G, Remy-Jardin M, Ryerson CJ, Myers JL, Kreuter M, Vasakova M, Bargagli E, Chung JH, Collins BF, Bendstrup E (2020). Diagnosis of hypersensitivity pneumonitis in adults an official ATS/JRS/ALAT clinical practice guideline. Am J Respir Crit Care Med.

[10] Hebert J, Beaudoin J, Laviolette M, Beaudoin R, Belanger J, Cormier Y (1985). Absence of correlation between the degree of alveolitis and antibody-levels to micropolysporum-faeni. Clin Exp Immunol.

[11] Mohr LC (2004). Hypersensitivity pneumonitis. Curr Opin Pulm Med.

[12] Joshi AD, Fong DJ, Oak SR, Trujillo G, Flaherty KR, Martinez FJ, Hogaboam CM (2009). Interleukin-17-mediated immunopathogenesis in experimental hypersensitivity pneumonitis. Am J Respir Crit Care Med.

[13] Rangel-Moreno J, Hartson L, Navarro C, Gaxiola M, Selman M, Randall TD (2006). Inducible bronchus-associated lymphoid tissue (iBALT) in patients with pulmonary complications of rheumatoid arthritis. J Clin Investig.

[14] Huppe CA, Blais Lecours P, Lechasseur A, Gendron DR, Lemay AM, Bissonnette EY, Blanchet MR, Duchaine C, Morissette MC, Rosen H, Marsolais D (2018). A sphingosine-1-phosphate receptor 1 agonist inhibits tertiary lymphoid tissue reactivation and hypersensitivity in the lung. Mucosal Immunol.

[15] Marin ND, Dunlap MD, Kaushal D, Khader SA (2019). Friend or Foe: the protective and pathological roles of inducible bronchus-associated lymphoid tissue in pulmonary diseases. J Immunol.

[16] McSharry C (2003). B lymphocytes in allergic alveolitis. Clin Exp Allergy.

[17] Vasakova M, Selman M, Morell F, Sterclova M, Molina-Molina M, Raghu G (2019). Hypersensitivity pneumonitis: current concepts of pathogenesis and potential targets for treatment. Am J Respir Crit Care Med.

[18] Tamm AM, Kremens K (2019). Rituximab for salvage therapy of refractory hypersensitivity pneumonitis. WMJ.

[19] Lota HK, Keir GJ, Hansell DM, Nicholson AG, Maher TM, Wells AU, Renzoni EA (2013). Novel use of rituximab in hypersensitivity pneumonitis refractory to conventional treatment. Thorax.

[20] Ferreira M, Borie R, Crestani B, Rigaud P, Wemeau L, Israel-Biet D, Leroy S, Quetant S, Plantier L, Dalphin JC (2020). Efficacy and safety of rituximab in patients with chronic hypersensitivity pneumonitis (cHP): a retrospective, multicentric, observational study. Respir Med.

[21] Carter S, Miard S, Caron A, Salle-Lefort S, St-Pierre P, Anhe FF, Lavoie-Charland E, Blais-Lecours P, Drolet MC, Lefebvre JS (2018). Loss of OcaB prevents age-induced fat accretion and insulin resistance by altering B-lymphocyte transition and promoting energy expenditure. Diabetes.

[22] Shakya A, Goren A, Shalek A, German CN, Snook J, Kuchroo VK, Yosef N, Chan RC, Regev A, Williams MA, Tantin D (2015). Oct1 and OCA-B are selectively required for CD4 memory T cell function. J Exp Med.

[23] Jankovic M, Nussenzweig MC (2003). OcaB regulates transitional B cell selection. Int Immunol.

[24] Huppe CA, Blais-Lecours P, Bernatchez E, Lauzon-Joset JF, Duchaine C, Rosen H, Dion G, McNagny KM, Blanchet MR, Morissette MC, Marsolais D (2020). S1P(1) contributes to endotoxin-enhanced B-cell functions involved in hypersensitivity pneumonitis. Am J Respir Cell Mol Biol.

[25] Israel-Assayag E, Fournier M, Cormier Y (1999). Blockade of T cell costimulation by CTLA4-Ig inhibits lung inflammation in murine hypersensitivity pneumonitis. J Immunol.

[26] Simonian PL, Roark CL, Wehrmann F, Lanham AK, del Valle FD, Born WK, O'Brien RL, Fontenot AP (2009). Th17-polarized immune response in a murine model of hypersensitivity pneumonitis and lung fibrosis. J Immunol.

[27] Lecours PB, Duchaine C, Taillefer M, Tremblay C, Veillette M, Cormier Y, Marsolais D (2011). Immunogenic properties of archaeal species found in bioaerosols. PLoS ONE.

[28] Thangada S, Khanna KM, Blaho VA, Oo ML, Im DS, Guo CY, Lefrancois L, Hla T (2010). Cell-surface residence of sphingosine 1-phosphate receptor 1 on lymphocytes determines lymphocyte egress kinetics. J Exp Med.

[29] Allende ML, Tuymetova G, Lee BG, Bonifacino E, Wu YP, Proia RL (2010). S1P1 receptor directs the release of immature B cells from bone marrow into blood. J Exp Med.

[30] Matloubian M, Lo CG, Cinamon G, Lesneski MJ, Xu Y, Brinkmann V, Allende ML, Proia RL, Cyster JG (2004). Lymphocyte egress from thymus and peripheral lymphoid organs is dependent on S1P receptor 1. Nature.

[31] Eken A, Duhen R, Singh AK, Fry M, Buckner JH, Kita M, Bettelli E, Oukka M (2017). S1P1 deletion differentially affects TH17 and Regulatory T cells. Sci Rep.

[32] Shiow LR, Rosen DB, Brdickova N, Xu Y, An J, Lanier LL, Cyster JG, Matloubian M (2006). CD69 acts downstream of interferon-alpha/beta to inhibit S1P1 and lymphocyte egress from lymphoid organs. Nature.

[33] Yoshizaki A, Iwata Y, Komura K, Ogawa F, Hara T, Muroi E, Takenaka M, Shimizu K, Hasegawa M, Fujimoto M (2008). CD19 regulates skin and lung fibrosis via Toll-like receptor signaling in a model of bleomycin-induced scleroderma. Am J Pathol.

[34] Poole JA, Mikuls TR, Duryee MJ, Warren KJ, Wyatt TA, Nelson AJ, Romberger DJ, West WW, Thiele GM (2017). A role for B cells in organic dust induced lung inflammation. Respir Res.

[35] Vandoorne K, Addadi Y, Neeman M (2010). Visualizing vascular permeability and lymphatic drainage using labeled serum albumin. Angiogenesis.

[36] Rice JC, Bucy RP (1995). Differences in the degree of depletion, rate of recovery, and the preferential elimination of naive CD4(+) t-cells by anti-CD4 monoclonal-antibody (GK1.5) in young and aged mice. J Immunol.

[37] De Sadeleer LJ, Hermans F, De Dycker E, Yserbyt J, Verschakelen JA, Verbeken EK, Verleden GM, Wuyts WA (2019). Effects of corticosteroid treatment and antigen avoidance in a large hypersensitivity pneumonitis cohort: a single-centre cohort study. J Clin Med.

[38] Amanna IJ, Carlson NE, Slifka MK (2007). Duration of humoral immunity to common viral and vaccine antigens. N Engl J Med.

[39] Zimmermann P, Curtis N. Factors that influence the immune response to vaccination. Clin Microbiol Rev. 2019; 32. 10.1128/CMR.00084-18 PMC6431125 30867162

[40] Allie SR, Randall TD (2020). Resident memory B cells. Viral Immunol.

[41] Allie SR, Bradley JE, Mudunuru U, Schultz MD, Graf BA, Lund FE, Randall TD (2019). The establishment of resident memory B cells in the lung requires local antigen encounter. Nat Immunol.

[42] Bice DE, Muggenburg BA (1996). Pulmonary immune memory: Localized production of antibody in the lung after antigen challenge. Immunology.

[43] Bice DE, Weissman DN, Muggenburg BA (1991). Long-term maintenance of localized antibody-responses in the lung. Immunology.

[44] Marsolais D, Hahm B, Walsh KB, Edelmann KH, McGavern D, Hatta Y, Kawaoka Y, Rosen H, Oldstone MBA (2009). A critical role for the sphingosine analog AAL-R in dampening the cytokine response during influenza virus infection. Proc Natl Acad Sci USA.

[45] Walsh KB, Marsolais D, Welch MJ, Rosen H, Oldstone MBA (2010). Treatment with a sphingosine analog does not alter the outcome of a persistent virus infection. Virology.

[46] Li XC, Ding Y, Zi MT, Sun L, Zhang WJ, Chen S, Xu YK (2017). CD19, from bench to bedside. Immunol Lett.

[47] Schuyler M, Gott K, Haley P (1991). Experimental murine hypersensitivity pneumonitis. Cell Immunol.

[48] Schuyler M, Gott K, Edwards B (1996). Experimental hypersensitivity pneumonitis: cellular requirements. Clin Exp Immunol.

[49] Melet J, Mulleman D, Goupille P, Ribourtout B, Watier H, Thibault G (2013). Rituximab-induced T cell depletion in patients with rheumatoid arthritis association with clinical response. Arthritis Rheum.

[50] Costabel U, Miyazaki Y, Pardo A, Koschel D, Bonella F, Spagnolo P, Guzman J, Ryerson CJ, Selman M (2020). Hypersensitivity pneumonitis. Nat Rev Dis Primers.

[51] Ando M, Konishi K, Yoneda R, Tamura M (1991). Difference in the phenotypes of bronchoalveolar lavage lymphocytes in patients with summer-type hypersensitivity pneumonitis, farmers lung, ventilation pneumonitis, and bird fanciers lung—report of a nationwide epidemiologic-study in Japan. Journal of Allergy and Clinical Immunology.

[52] Morais A, Winck JC, Delgado L, Palmares MC, Fonseca J, Sa JME, Marques JA (2004). Suberosis and bird fancier’s disease: a comparative study of radiological, functional and bronchoalveolar lavage profiles. J Investig Allergol Clin Immunol.

[53] Selman M, Lacasse Y, Pardo A, Cormier Y (2010). Hypersensitivity pneumonitis caused by fungi. Proc Am Thorac Soc.

[54] Selman M, Pardo A, King TE (2012). Hypersensitivity pneumonitis insights in diagnosis and pathobiology. Am J Respir Crit Care Med.

