# Supplementary material for: Co-modulation of T cells and B cells enhances the inhibition of inflammation in experimental hypersensitivity pneumonitis
Source: Respir Res. 2022 Oct 8;23:275. doi: 10.1186/s12931-022-02200-9 (PMC9547367; doi:10.1186/s12931-022-02200-9)
Supplement: Supplementary file 1 — Additional file 1. Additional details relating to leukocyte subset redistribution and activation. Figure S1. Adoptively transferred B cells minimally accumulate in Rag1−/− mice lungscontrarily to T cells. Figure S2. Unaltered numbers of macrophages and DCs in the lung of mice with CD19-drivenS1P1 deletion. Figure S3. Effect of CD19-driven S1P1 deletion on archetypal HP-associated CD4 T cell responsesin the lung. Figure S4. Impact of CD19-driven S1P1 deletion on mLN DC and CD4 T cells. [file 12931_2022_2200_MOESM1_ESM.docx]

**SUPPLEMENTAL DATA**

**
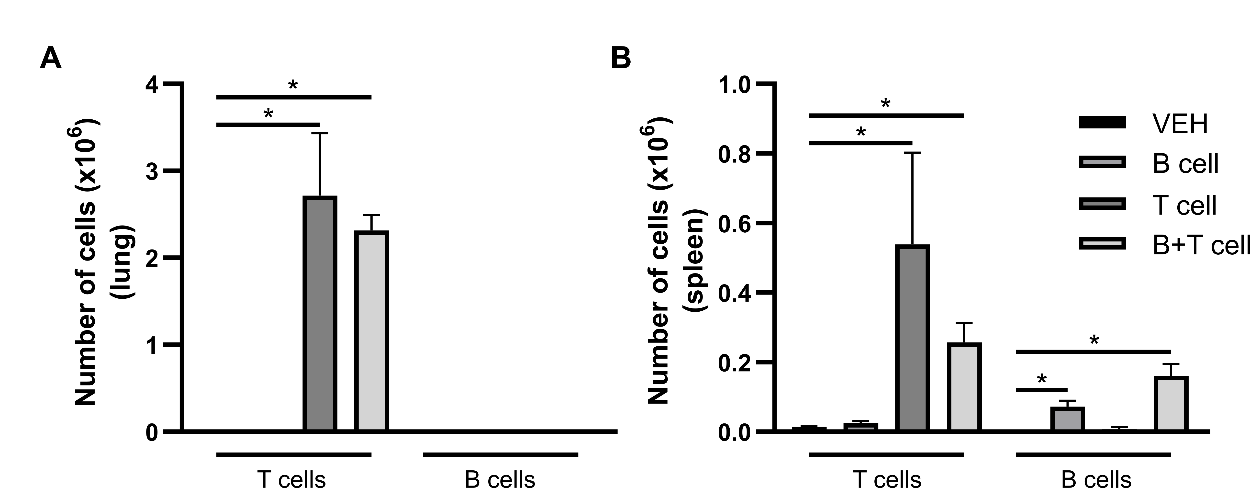
**

**Figure S1: Adoptively transferred B cells minimally accumulate in Rag1^-/-^ mice lungs contrarily to T cells.** Saline, 5 x 10^6^ B cells, 2.5 x 10^6^ T cells, or the combination of 5 x 10^6^ B cells and 2.5 x 10^6^ T cells were injected intravenously to Rag1-deficient mice. Beginning 48h after the transfer, mice were exposed i.n. three consecutive days a week for three weeks to 100µg MSS and euthanized 24 hours after the last exposure. Flow cytometry was performed on single cell suspensions to assess the total numbers of B cells (CD19^+^CD90^-^) and T cells (CD19^-^CD90^+^CD4^+^ and CD19^-^CD90^+^CD8^+^) present in **(A)** the lung and **(B)** the spleen.


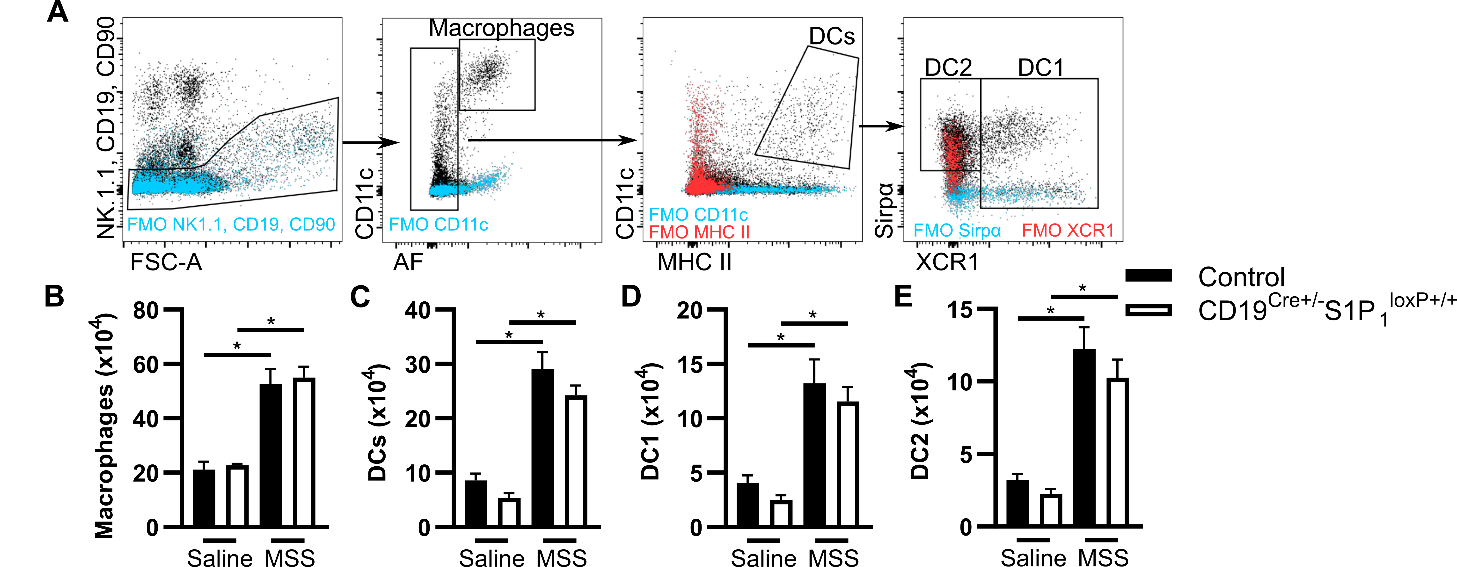


**Figure S2: Unaltered numbers of macrophages and DCs in the lung of mice with CD19-driven S1P_1_ deletion.** Control and CD19^Cre+/-^ S1P_1_^loxP+/+^ mice were exposed three consecutive days a week for three weeks to saline or 100µg MSS and euthanized 24 h after the last exposure. **A)** Gating strategy. NK1.1^+^, CD19^+^ and CD90^+^ cells were discarded, and macrophages were identified as NK1.1- CD19- CD90.2- CD11c^hi^ AF^+^ cells. DCs were selected as NK1.1^-^ CD19^-^ CD90.2^-^ AF^low^ CD11c^+^ MHCII^+^ cells. From the total DC population, DC1s were identified as XCR1^+^ and DC2s as Sirpα^+^ XCR1^-^ DCs. Frequencies of macrophages and DC subsets were multiplied by the total number of lung cells to determine the absolute numbers of **(B)** lung macrophages, and **(C-E)** total DCs and DC subsets. AF: Autofluorescence. DC: Dendritic cells. FMO: Fluorescence minus one control. MSS: *Methanosphaera stadtmanae*. Averages ± SEM. n=10-11. * p<0.05.

**
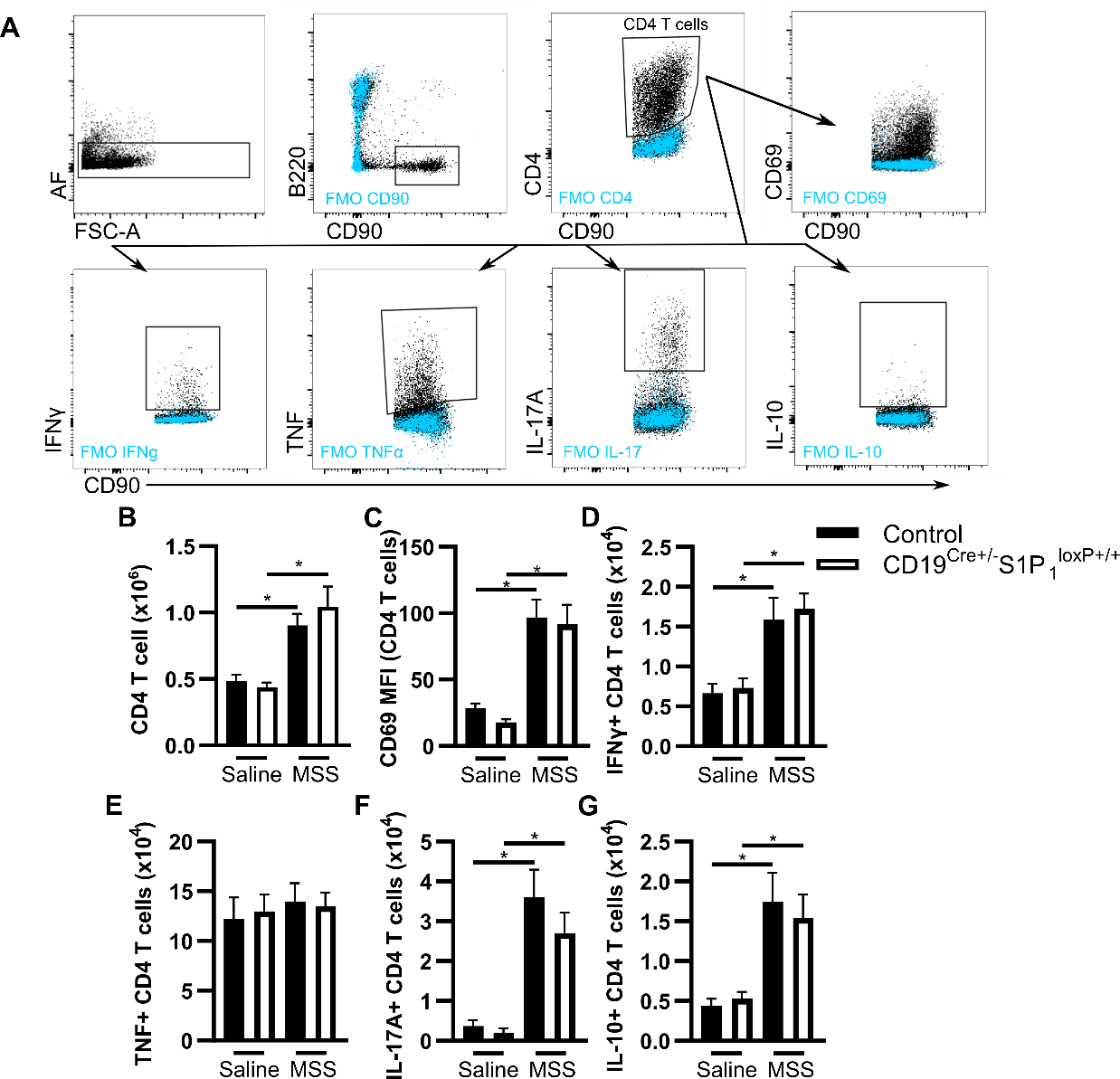
**

**Figure S3: Effect of CD19-driven S1P_1_ deletion on archetypal HP-associated CD4 T cell responses in the lung.** Control and CD19^Cre+/-^ S1P_1_^loxP+/+^ mice were instilled with saline or MSS as described in the methods section and euthanized 24 h after the last instillation. Intracellular cytokines labeling was done after 4 hours of stimulation with PMA (50 ng/ml), ionomycin (500 ng/ml), and brefeldin A (10 µg/ml) in staining buffer supplemented with 0.2% saponin for 20 minutes after fixation with 2% formaldehyde for another 20 minutes at 4°C. **A)** Gating strategy. From the SSC^low^ FSC^low^ population, CD4 T cells were identified as AF^-^B220^-^CD90^+^CD4^+^ cells. **B)** Total CD4 T cells, **(C)** CD69 MFI on CD4 T cells, and numbers of **(D)** IFNγ^+^, **(E)** TNF^+^, **(F)** IL‑17A^+^ and **(G)** IL‑10^+^ CD4 T cells were computed. AF: Autofluorescence. FMO: Fluorescence minus one control. MFI: Median fluorescence intensity. MSS: *Methanosphaera stadtmanae*. Averages ± SEM. n=5‑11. * p<0.05.


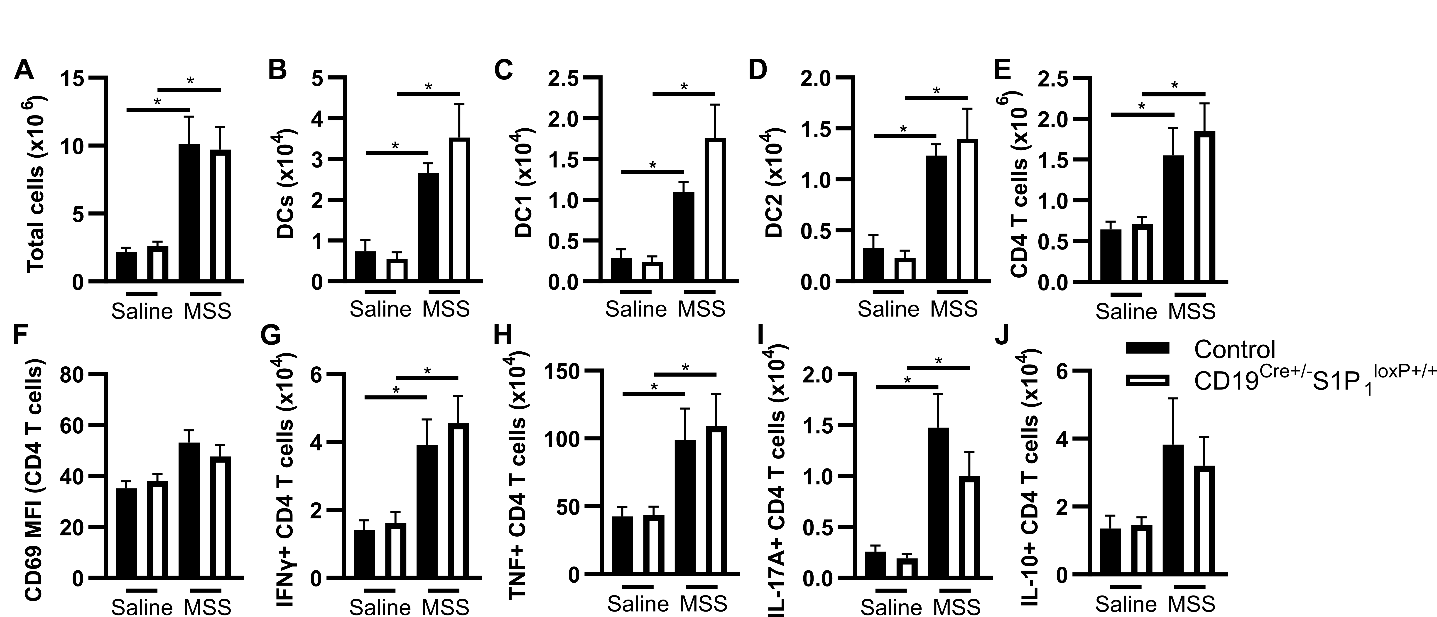


**Figure S4: Impact of CD19-driven S1P_1_ deletion on mLN DC and CD4 T cells**. Mice were exposed to saline or MSS as described in methods and euthanized 24h after the last instillation. Cell populations were identified using flow cytometry gating strategies described in methods and figures S1 and S2. **A)** Total mLN cell counts and numbers. Numbers of total **(B)** DCs, **(C)** DC1s**,** **(D)** DC2s, and **(E)** CD4 T cells are shown. **F)** CD69 MFI on CD4 T cells and numbers of **(G)** IFNγ^+^, **(H)** TNF^+^, **(I)** IL‑17A^+^, and **(J)** IL‑10^+^ CD4 T cells were computed. DC: Dendritic cells. MFI: Median fluorescence intensity. MSS: *Methanosphaera stadtmanae*. Averages ± SEM. n=5‑11. * p<0.05.
